# The influence of age, sex, and exercise on autophagy, mitophagy, and lysosome biogenesis in skeletal muscle

**DOI:** 10.1186/s13395-022-00296-7

**Published:** 2022-06-11

**Authors:** Matthew Triolo, Ashley N. Oliveira, Rita Kumari, David A. Hood

**Affiliations:** 1 School of Kinesiology and Health Science, Muscle Health Research Centre, York University, Toronto, Ontario M3J 1P3 Canada; 2 Muscle Health Research Centre, York University, Toronto, Ontario M3J 1P3 Canada

**Keywords:** Autophagy, Mitophagy, Lysosomes, Muscle, Sex differences, Aging, TFEB

## Abstract

**Background:**

Aging decreases skeletal muscle mass and quality. Maintenance of healthy muscle is regulated by a balance between protein and organellar synthesis and their degradation. The autophagy-lysosome system is responsible for the selective degradation of protein aggregates and organelles, such as mitochondria (i.e., mitophagy). Little data exist on the independent and combined influence of age, biological sex, and exercise on the autophagy system and lysosome biogenesis. The purpose of this study was to characterize sex differences in autophagy and lysosome biogenesis in young and aged muscle and to determine if acute exercise influences these processes.

**Methods:**

Young (4–6 months) and aged (22–24 months) male and female mice were assigned to a sedentary or an acute exercise group. Mitochondrial content, the autophagy-lysosome system, and mitophagy were measured via protein analysis. A TFEB-promoter-construct was utilized to examine Tfeb transcription, and nuclear-cytosolic fractions allowed us to examine TFEB localization in sedentary and exercised muscle with age and sex.

**Results:**

Our results indicate that female mice, both young and old, had more mitochondrial protein than age-matched males. However, mitochondria in the muscle of females had a reduced respiratory capacity. Mitochondrial content was only reduced with age in the male cohort. Young female mice had a greater abundance of autophagy, mitophagy, and lysosome proteins than young males; however, increases were evident with age irrespective of sex. Young sedentary female mice had indices of greater autophagosomal turnover than male counterparts. Exhaustive exercise was able to stimulate autophagic clearance solely in young male mice. Similarly, nuclear TFEB protein was enhanced to a greater extent in young male, compared to young female mice following exercise, but no changes were observed in aged mice. Finally, TFEB-promoter activity was upregulated following exercise in both young and aged muscle.

**Conclusions:**

The present study demonstrates that biological sex influences mitochondrial homeostasis, the autophagy-lysosome system, and mitophagy in skeletal muscle with age. Furthermore, our data suggest that young male mice have a more profound ability to activate these processes with exercise than in the other groups. Ultimately, this may contribute to a greater remodeling of muscle in response to exercise training in males.

## Background

The natural aging process is associated with a progressive loss of muscle mass and function [1, 2], commonly referred to as sarcopenia [3]. Since skeletal muscle represents 40% of total body mass and is essential for motor function and whole-body metabolic control [1, 4], these age-related declines are associated with deficits in the quality of life of older individuals and are related to a higher incidence of falls, hospitalization, and co-morbidities [5]. This is problematic when we consider that physical inactivity rates are greater in those that are older, potentiating the negative effects of age on overall health [6–8]. It is now known that the maintenance of physical activity throughout the lifespan is an essential preventative measure in age-associated loss in mitochondrial volume and function [9, 10]. Thus, there is an evolving need to understand the mechanisms that underly the changes in muscle architecture with age, and how exercise preserves muscle health.

The muscle atrophy observed within the aging muscle is achieved by an imbalance protein synthesis and degradation. The autophagy-lysosome system is a proteolytic pathway that is responsible for the breakdown of long-lived, aggregated proteins and organelles [11]. Autophagy is an evolutionary conserved recycling mechanism, whereby damaged or dysfunctional cellular components are engulfed in a double membrane autophagosome and delivered to the lysosomes for digestion. Inhibition of autophagy promotes atrophy, neuromuscular junction decay, sarcomere disarrangement, and ultimately weakness [12–16]. Furthermore, a lack of autophagy attenuates the phenotypic remodeling of muscle associated with exercise training [17–19]. Cumulatively, these studies highlight the importance of this proteolytic system in skeletal muscle.

The impact of aging on skeletal muscle autophagy remains controversial [20], but previous reports utilizing “flux” measurements have shown that autophagy is upregulated in aging muscle [21, 22]. These alterations in autophagy have implications for the selective degradation of mitochondria through mitophagy. In fact, in a series of studies, our group has reported enhanced mitophagy in aged muscle [22–24]. We have also shown that muscle from aged rodents displayed an accumulation of lysosomal protein and nondegraded lysosomal content, termed lipofuscin [22, 23]. This would imply lysosomal dysregulation, which may contribute to a reduced capacity to effectively remove damaged intracellular constituents.

In young, healthy muscle, autophagy and lysosome biogenesis are activated following acute endurance activity [25, 26] to assist in the remodeling of muscle. Over time, aerobic training improves the metabolic capacity of the tissue [27–29], enhancing mitochondrial content concomitant with increases in lysosomal content [30, 31]. However, it remains to be seen if these acute-exercise responses occur in aged skeletal muscle, and whether exercise can enhance lysosome capacity to promote the removal of the accumulating damaged constituents.

Recently, it was reported that female mice had enhanced catabolic and autophagy signaling in response to hindlimb unloading [32, 33]. These findings highlight the importance of examining biological sex as a variable in muscle physiology. Furthermore, a limited analysis on the autophagy-lysosome markers was conducted in aged male and female mice, investigating the impact of prolonged training in these groups. No difference between the sexes was reported [34]. We are unaware of any studies that have examined the influence of biological sex on the autophagy-lysosome system, with a focus on the impact of age and acute exercise.

Thus, the overarching goal of this study was to examine sex differences in autophagic, mitophagic, and lysosomal pathways in muscle from young (4–6 months) and aged (22–24 months) male and female C57BL6 mice. Based on previous reports [32, 33], we hypothesized that autophagy would be greater in our female cohort. Furthermore, we investigated the utility of acute exercise to activate these pathways in young and aged muscle and whether biological sex could influence the exercise response.

## Materials and methods

### Animals

All animal procedures were conducted in accordance with the standards set by the Canadian Council on Animal Care, with the approval of the York University Animal Care Committee (YUACC). Young (4–6 months) and aged (22–24 months) male and female C57BL/6 mice were obtained from The Jackson Laboratory. Mice used in this study were ordered at ~2 months old and aged in our facility in accordance with YUACC protocols and guidelines. Food and water were provided ad libitum. At the appropriate age, mice were assigned to sedentary or acute exhaustive exercise groups so that the final # of animals/group were *n* = 5/ male; *n* = 4/female groups.

### Acute exhaustive exercise protocol

Animals that were assigned to the acute exhaustive exercise group were acclimatized to the treadmill 48 and 24 h prior to their exercise date. Acclimatization occurred at 0m/min, 5m/min, and 10m/min for 5 min each. On the day of exercise, prior to protocol, resting blood lactate levels were measured via tail blood. Subsequently, animals were placed on the treadmill at a fixed incline of 10%. The acute exhaustive exercise protocol began with a 5m/min warmup for 5 min and a 10m/min run for 10 min, followed by increasing speeds at 1m/min every 2 min until exhaustion was achieved. Exhaustion was defined as the inability of the animal to run on the treadmill despite prodding. Immediately following exercise, post-exercise blood lactate measurements were made, animals were cervically dislocated and tissues were harvested for biochemical analysis.

### Luciferase reporter assay

The TFEB promoter containing −1601-bp region of the canonical promoter was subcloned into a pGL3 vector containing a firefly luciferase reporter (rTFEB-pGL3) [26]. Ampicillin-resistant bacteria were transformed, and bacterial colonies were then amplified to isolate plasmid DNA using a Maxi Plasmid Isolation Kit (Qiagen). Six days prior to tissue removal, in both the sedentary and exercised groups, mice underwent in vivo muscle transfection. Briefly, mice were anesthetized using gaseous isoflurane and the lower hindlimbs were shaved and sterilized. One gastrocnemius muscle of each mouse was injected with 30μg of the rTFEB-pGL3 construct and 50ng of Renilla luciferase downstream of the CMV promoter (pRL-CMV), used as a marker of transfection efficiency. The contralateral hindlimb was injected with an empty vector (pGL3) and Renilla luciferase, both under the control of the CMV promoter. All injections were conducted using a short 29-gage insulin syringe (BD Canada). Immediately after the injection, trans-continuous electrical pulses were applied using an ECM 380 BTX electroporation system (Harvard Apparatus Saint-Laurent, QC, Canada), whereby the muscle was held on either side of the injection site by forceps-style electrodes, followed by ten 100V/cm^2^ pulses. The conductive gel was applied to the electrodes to assist with transfection. The anode and cathode orientation were reversed, and another 10 pulses were delivered. Following tissue extraction, frozen gastrocnemius muscle was pulverized to a fine powder at the temperature of liquid nitrogen. Approximately 30mg of powder was diluted in 1X passive lysis buffer (Promega, cat# E1500) supplemented with protease (Roche Mississauga, ON, Canada) and phosphatase (Sigma Oakville, ON, Canada) inhibitors. The sample was then sonicated on ice (3×3s) and spun in a microcentrifuge at 4°C for 10 min at 16,000g. The supernatant fraction was then collected and luciferace activty was measured using an EG&G Berthold Luminometer (Lumat LB 7507; Berhold Technologies, Oak Ridge, TN). Following initial background readings of the passive lysis buffer, 20μL of either the rTFEB-PGL3+ pRL-CMV or pGL3+pL-CMV sample tissue was loaded into a test tube and mixed with 100μl of luciferase substrate followed by 100μl of Renilla substrate (Promega). Each sample was run in triplicate, and the average was used. The ratio of firefly luciferase reporter (RLU1) to Renilla luciferase (RLU2) was taken for both the TFEB-promoter- and the empty vector-injected limbs. Transcriptional activity was expressed as the TFEB promoter data divided by the empty vector.

### High-resolution respiration and ROS-emission

High-resolution respirometry (Oroboros O2k, Austria) was used to measure oxygen consumption in permeabilized muscle fibers from the lateral portion of the left TA muscle of all mice. Briefly, the muscle was excised, and fibers were mechanically separated in ice cold BIOPS buffer (2.77mM CaK2EGTA, 7.23mM K2EGTA, 7.55mM Na2ATP, 6.56mM MgCl26H2O, 20mM Taurine, 15mM Na2Phosphocreatine, 20mM Imidazole, 0.5mM Dithiothreitol, 50mM MES-Hydrate, pH 7.1). Subsequently, the fibers were permeabilized in BIOPS supplemented with 40μg/μL saponin at 4°C for 30 min with gentle rocking and washed in Buffer-Z (105mM K-MES, 30mM KCl, 10mM KH2PO4, 5mM MgCl2•6H2O, 1mM EGTA, 5mg/ml BSA) with gentle rocking. Fibers were then incubated in the chamber with oxygenated Buffer-Z supplemented with 10μM Amplex-Red to simultaneously measure ROS production, as well as 1μM Blebbistatin to prevent tetanus of the muscle [35], 25U/ml Cu/Zn SOD1 to convert O2- to H2O2, and 2mM EGTA. Following oxygenation and measurement of background values, substrates were added to assess respiration and ROS production simultaneously. Substrates were titrated in three separate protocols as follows. In the first protocol, 5mM glutamate + 2mM malate (Complex I – Basal), 5mM ADP (Complex I – Active), and 10mM succinate (Complex I+II – Active) were added to simultaneously measure O_2_ consumption and ROS. In the second protocol, O_2_ consumption was measured by first titrating 0.5μM rotenone, to prevent electron backflow and slip at Complex I and damage to the fiber. Subsequently, 10mM succinate (Complex-II Basal) and 5mM ADP (Complex-II Active) were added. In the final protocol, ROS emission was measured by titrating 10mM of succinate (Complex-II Basal) and 5mM ADP (Complex-II Active). To test for mitochondrial membrane integrity, cytochrome c was added to the chamber. Respiratory function was first determined by calculating oxygen flux rates (pmol/s·ml) minus background rates and corrected to fiber mass (pmol/s/mg). Respiration was also normalized to protein from the OXPHOS data in Fig. 3 as follows: Complex-I Active respiration to Complex I protein, Complex-II Active respiration to Complex II protein, and Complex I and II Active respiration to total OXPHOS protein. ROS emission was calculated by dividing the rate of ROS emission (pmol/s/mg) and correcting it by the corresponding respiration rate (pmol H_2_O_2_/pmol O_2_ consumed).

### Cytosolic and nuclear fractionation

Nuclear and cytosolic fractions from fresh TA muscles of mice were obtained using the NE-PER extraction reagents (38835, Thermo Scientific Scientific) with minor modifications. Briefly, ~50-100mg of the TA muscle was minced on ice and homogenized using a Dounce homogenizer in cytosolic extraction reagent (CER) I. Homogenates were then vortexed and let to stand on ice for 10 min. Following the addition of CER II solution, samples were briefly vortexed and centrifuged (16,000g) for 10 min. The cytosolic fractions (supernates) were then collected. The remaining pellets, containing nuclei and cellular debris, were washed 3 times in cold 1×PBS and subsequently resuspended in nuclear extraction buffer (NER). Nuclear fractions were then sonicated (3x3s) and incubated on ice for 40 min. These samples were vortexed every 10 min during the incubation and subsequently underwent centrifugation (16,000g) for 10 min. The resulting supernatant nuclear fractions were collected. Both the cytosolic and nuclear fractions were stored at −80°C until further analysis.

### Whole muscle protein extracts

One quadricep muscle was snap frozen in liquid nitrogen following excision from the animal and stored at −80°C. The tissue was pulverized to a fine powder at the temperature of liquid nitrogen. Protein extracts were made by diluting (10×) a small amount of powder (~15–20mg) in Sakamoto buffer (20mM HEPES, 2mM EGTA, 1% Triton X-100, 50% Glycerol, 50 mM ß-Glycerophosphate) containing both phosphatase (Sigma) and protease (Roche) inhibitors and rotated end-over-end for 1 h at 4°C. Samples were then sonicated on ice (3x3s) and centrifuged (14,000g) for 15 min at 4°C. The supernatant fraction was collected and stored at −80°C until further analysis.

### Western blotting

All protein concentrations in nuclear and cytosolic fractions and whole muscle samples were determined using the Bradford method. Equal amounts of protein (~20–30μg) were loaded and separated via SDS-PAGE and transferred onto nitrocellulose membranes (Bio-Rad, Mississauga, ON, Canada). Membranes were blocked with wash buffer (0.12% Tris-HCl, 0.585% NaCl, 0.1% Tween, pH 7.5) supplemented with 5% skim milk (w/v) at room temperature for 1 h with gentle agitation. Membranes were then incubated with primary antibodies overnight at 4°C for OXPHOS Cocktail (Ab110413, Lot 2101000654, Abcam), Beclin1 (3738, Lot 3, Cell Signaling Technologies), ATG7 (A2856, Lot 078M4843V, Sigma), p62 (Ab56416, Lot GR3285986-1, Abcam), LC3-I/II (4108, Lot 3, Cell Signaling Technologies), Bnip3 (Gift from Dr. L.A. Kirshenbaum), Parkin (4211, Lot 7, Cell Signaling Technologies), VDAC (Ab14734, Lot GR3391163-2, Abcam), Lamp1 (Ab24170, Lot GR3235632-1, Abcam), V-ATPase B1/2 (sc-55544 F-6, Lot I1018, SantaCruz), mature Cathepsin B (D1C7Y, Lot 1, Cell Signaling Technologies), mature Cathepsin D (sc-377299, Lot BO419, SantaCruz), TFEB whole muscle (MBS120432, Lot 319C2a-3, MyBioSource), TFEB nuc/cyto (A303-673A, Lot 7, Bethyl), TFE3 (HPA023881, Lot 000010514, Sigma), GAPDH (ab8254, Lot GR3317834-1, Abcam), a-tubulin (CP06, Lot D00175772, Calbiochem), and H2B (2934, Lot 4, Cell Signaling Technologies). The following day, membranes were washed 3 × 5min in wash buffer and incubated for 1 h at room temperature with the appropriate HRP-conjugated secondary antibody and subsequently washed 3×5 min in wash buffer. The protein density was visualized using enhanced chemiluminescence (1705061, Bio-Rad) with an iBright FL1500 Imaging Station (Fischer Scientific, Oakville, ON, Canada). Band densities were quantified by ImageJ software (NIH) and normalized to corresponding loading controls. All gels were run in a sex-specific manner containing either male or female samples. For whole muscle western blots, to enable statistical comparisons between the sexes, an identical, arbitrary control sample was run in the first lane of all gels. All values were then normalized to this control band, represented as “C” in depicted blots. Representative blots with a break between bands denote that a portion of the image was shifted, without alterations in contrast, to show representative data in comparison to the arbitrary control band ran on that gel. All western blot data are represented as combined male and female for young and old groups and sex-separated data.

### Statistical analysis

Data were analyzed using GraphPad Prism Software (version 9) and values are represented as means ± SEM. Student’s unpaired *t*-tests were utilized to analyze combined, male and female data to investigate an effect of age. Two-way ANOVAs were used to assess the interaction between age and sex where applicable, and significance was achieved at *p* < 0.05. A Bonferroni post hoc test was used and *δ* represents a significant difference. Due to the limited sample sizes when the sexes were separated, where post hoc tests failed to uncover significant differences between groups, independent Student’s unpaired *t*-tests were used to assess the differences between male and female, young and old data. Significance is represented by *, and *p* values are shown for trends in the data. Three-way ANOVAs were used to assess the independent and interaction effects between age, sex, and exercise; significance was achieved at *p* < 0.05, and *p* values are reported for trends in the data set where applicable.

## Results

### Physical characteristics of young and aged male, and female mice

To determine whether aging differentially impacted muscle mass in male and female mice, we first measured body mass (g), muscle mass (mg), and muscle mass corrected for body mass (mg/g) in the predominantly fast tibialis anterior (TA) and predominantly slow-twitch soleus (Sol) muscles (Table 1). Overall, body mass was 1.4-fold greater in aged mice versus young counterparts (*p* < 0.05). Main effects of age and sex effects were found. Post hoc tests revealed that both young and aged male mice were significantly larger than age-matched female counterparts (*p* < 0.05). Furthermore, aged male mice were 32% heavier than young males, and aged female mice were 28% heavier than young females (*p* < 0.05). Raw TA mass (mg) was lower in female mice (sex effect, *p* < 0.05) and TA mass was significantly less in young females versus young males (*t*-test, *p* < 0.05). When corrected for body mass, TA mass was 24% smaller in aged mice (*p* < 0.05), and a main effect of sex was observed in our separated analysis (*p* < 0.05). On average, TA mass/body mass (mg/g) was 28% lower in female mice (post hoc, *p* < 0.05) and 18% less in male mice (*t*-test, *p* < 0.05). Sol mass (mg) was not different between any groups. When corrected for body mass (mg/g), a significant 30% decrease in Sol mass with age was measured in sex-pooled data (*p* < 0.05). In a sex-separated analysis, a main effect of age and sex was observed (*p* < 0.05). Young females had a 1.4-fold larger Sol mass/body mass than young males (post hoc, *p* < 0.05). With age, male mice had a 24% decline in Sol mass/body mass (*t*-test, *p* < 0.05), whereas females displayed a 36% decline (post hoc, *p* < 0.05).

**Table 1:** Animal body weight and muscle characteristics

	Combined		Male		Female		Statistics
	Young	Aged	Young	Aged	Young	Aged	
**Body mass (g)**	30.73 ± 1.27	41.35 ± 1.91^ϕ^	34.65 ± 1.01	45.72 ± 2.45^*,A^	25.83 ± 0.976^C^	35.89 ± 1.62^*,B,D^	# †
**Muscle mass (mg)**							
**TA**	51.41 ± 1.41	53.49 ± 3.95	54.61 ± 1.34	58.70 ± 3.95	47.43 ± 1.97^δ^	46.98 ± 5.58	†
**Sol**	10.32 ± 0.49	9.60 ± 0.76	10.01 ± 0.42	9.95 ± 0.926	10.51 ± 1.00	9.15 ± 1.32	
**Muscle mass (mg/g body weight)**							
**TA**	1.70 ± 0.06	1.31 ± 0.10^ϕ^	1.59 ± 0.05	1.30 ± 0.12^*^	1.85 ± 0.08	1.34 ± 0.17^*,B^	#
**Sol**	0.34 ± 0.03	0.24 ± 0.02^ϕ^	0.29 ± 0.02	0.22 ± 0.02^*^	0.41 ± 0.04^C^	0.26 ± 0.04^*,B^	# †

Body mass (g), muscle mass (mg), and muscle mass corrected for body weight (mg/g) in combined and sex-separated young and aged muscle. Values are means ± SEM. ϕ, *p* < 0.05, *t*-test between young and old in the combined analysis. In the sex-separated analysis, a two-way ANOVA was performed. #, *p* < 0.05 main effect of age; †, *p* < 0.05 main effect of sex; ⁋, *p* < 0.05 interaction of age and sex. “A” represents post hoc significance between young and aged within the male cohort, “B” represents post hoc significance between young and aged within the female cohort, “C” represents post hoc significance between young males and females, and “D” represents post hoc significance between aged males and females. Additionally, independent *t*-tests were run to compare young vs old within the same sex, *p < 0.05, and between male and females within the same age group, δ, *p* < 0.05. *N* = 10/male group, *N* = 8/female group

### Exercise capacity in young and aged, male and female mice

**Fig. 1:**
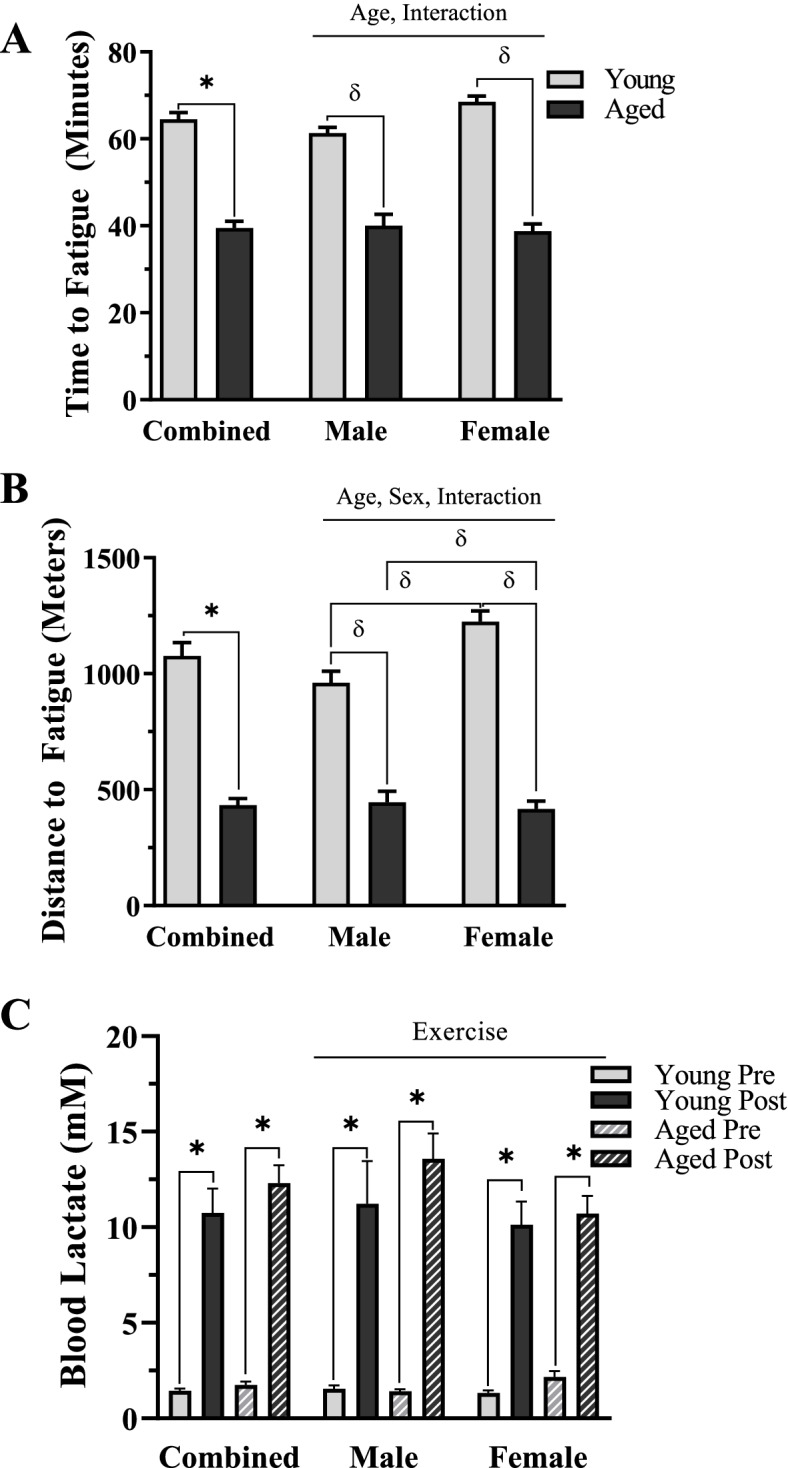
Exercise capacity in young and aged, male and female mice. **A** Time to fatigue in minutes. **B** Distance to fatigue in meters. **C** Blood lactate (mM). Values are means ± SEM. The main effects are represented on a graph at *p* < 0.05. *p < 0.05, *t*-test between indicated groups. δ *p* < 0.05, post hoc significance. *N* = 5/male group, *N* = 4/female group

To determine if age and biological sex impact acute exercise capacity, we exposed a cohort of mice to an exhaustive bout of incremental exercise. In our sex-pooled comparison, aged mice ran for an average of 25 min less (*t*-test, *p* < 0.05, Fig. 1A) accounting for 645 m of less distance covered (*t*-test, *p* < 0.05, Fig. 1B). In sex-separated comparisons, a main effect of age and an interaction of age and sex were found in run time (*p* < 0.05, Fig. 1A). Further analysis revealed 34% and 43% declines in aged male and female mice versus their young counterparts, respectively (post hoc, *p* < 0.05, Fig. 1A). Effects of age, sex, and an interaction of the two variables were measured in distance to fatigue (*p* < 0.05, Fig. 1B). Run distance was reduced with age in both male and female mice (post hoc, *p* < 0.05, Fig. 1B). On average, young female mice ran 263 m more than young males (post hoc, *p* < 0.05, Fig. 1B), whereas aged females ran slightly less (28m) than aged males (post hoc, *p* < 0.05, Fig. 1B). Blood lactate was similarly increased with exercise in all groups (*t*-test, *p* < 0.05, Fig. 1C).

### Mitochondrial parameters in young and aged, male and female mice

**Fig. 2:**
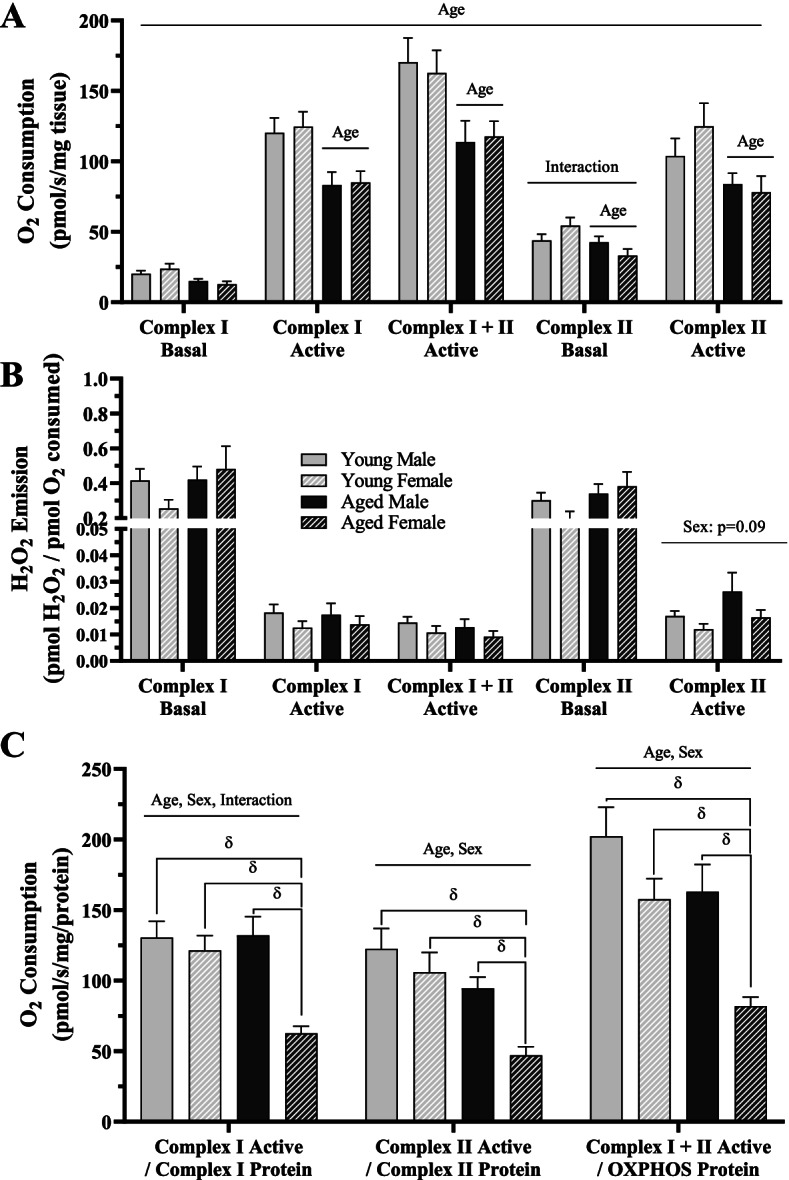
Mitochondrial respiration and reactive oxygen species in young and age, male and female mice. **A** Oxygen consumption rates, **B** H_2_O_2_ emission, and **C** oxygen consumption rates normalized to mitochondrial protein content in the indicated respiratory states. All values are reported as means ± SEM. For **A** and **B**, the main effects of a 3-way ANOVA are represented on the graph at *p* < 0.05. For **A**–**C**, the main effects of 2-way ANOVA are represented on the graph at *p* < 0.05. δ *p* < 0.05, post hoc significance. *N* = 10/male group, *N* = 8/female group

To understand the divergent endurance capacity with age and sex, we assessed mitochondrial parameters as these organelles are correlated with muscle fatigability. We examined respiration and H_2_O_2_ emission in permeabilized TA muscle fibers from all groups (Fig. 2A, B). We observed an overall effect of age, whereby aged muscle had lower respiratory capacity (3-way ANOVA, *p* < 0.05, Fig. 2A). Independent analyses were performed for each subsequent titration, and we measured a main effect of age for all respiratory measurements (2-way ANOVA, *p* < 0.05 Fig. 2A), apart from the Complex I-Basal condition. An interaction between age and sex was found in Complex II-Basal respiration (2-way ANOVA, *p* < 0.05 Fig. 2A); however, no post hoc significance was observed. Overall, no changes were measured in H_2_O_2_ emission in permeabilized fibers (Fig. 2B), but a trending effect of sex was measured in Complex II-active (2-way ANOVA, *p* = 0.09, Fig. 2B), with lower values in female samples.

**Fig. 3:**
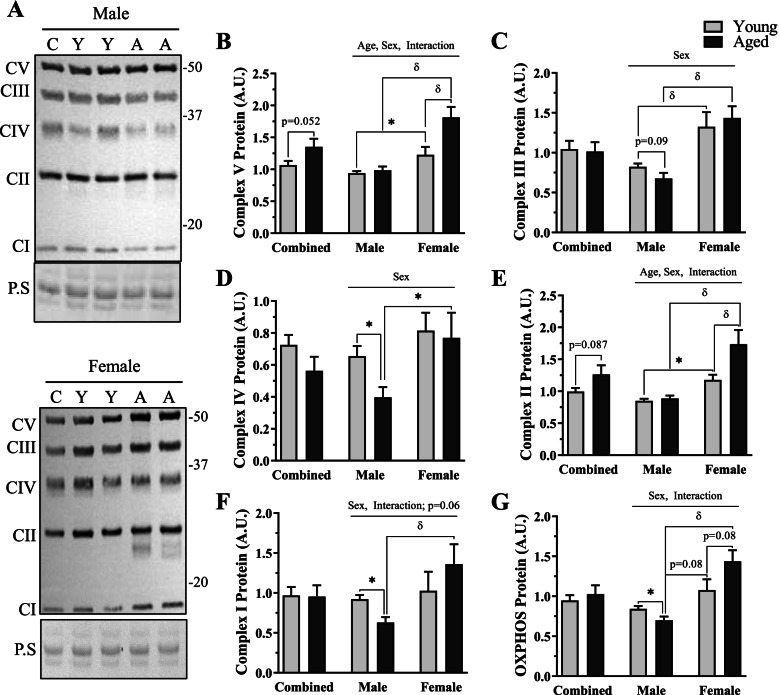
Mitochondrial protein content in young and age, male and female mice. **A** Representative western blot from male (top panel) and female (bottom panel) mice for OXPHOS protein. **B**–**F** Quantification of each independent mitochondrial protein. **B** Complex-V protein (ATP5A) protein, **C** Complex-III (UQCRC2) protein, **D** Complex-IV (MTCO1) protein, **E** Complex-II (SDH8), and **F** Complex I (NDUFB8) protein. **G** Quantification of total OXPHOS. All values were corrected to Ponceau stain (P.S), and values are reported as means ± SEM, in A.U. The main effects of 2-way ANOVA are represented on the graph at *p* < 0.05. δ *p* < 0.05, post hoc significance. *p < 0.05, *t*-test between indicated groups. *N* = 10/male group, 8/female group

To determine the effects of age and sex on mitochondrial protein content, we quantified levels of proteins derived from each complex of the electron transport chain (ETC) (Fig. 3). In the sex-grouped data, we found no significant differences in any ETC proteins, and a trending increase in both Complex-V (*t*-test, *p* = 0.058, Fig. 3B) and Complex-II protein (*t*-test, *p* = 0.087, Fig. 3B, E). A main effect of age was observed in both Complex-V (Fig. 3B) and Complex-II (Fig. 3E). Each independent complex (Fig. 3B–F) and total OXPHOS protein (Fig. 3G) exhibited a main effect of sex (*p* < 0.05), such that females had more mitochondrial protein. Furthermore, an interaction between age and sex was found in Complex-V (*p* < 0.05, Fig. 3B), Complex-II (*p* < 0.05, Fig. 3E), Complex-I (*p* < 0.05, Fig. 3F), and total OXHOS (*p* < 0.05, Fig. 3G) protein, whereby female muscle did not display decrements in mitochondrial protein content with age.

We assessed independent differences between young males and females and measured 35%, 61%, 38%, and 28% more Complex-V (*t*-test, *p* < 0.05, Fig. 3B), Complex-III (post hoc, *p* < 0.05, Fig. 3C), Complex-II (*t*-test, *p* < 0.05, Fig. 3E), and total OXPHOS (*t*-test, *p* = 0.08, Fig 3G) protein in young females versus young males, respectively. The same comparison in aged male and female mice showed that each complex had between 1.8- and 2.1-fold more mitochondrial protein (*p* < 0.05, Fig. 3B–F) and 2.1-fold more total OXPHOS in females than in males (post hoc, *p* < 0.05, Fig. 3G).

We then explored independent differences between young and aged muscle from same-sex mice. In male mice, we observed no change in Complex-V (Fig. 3B) or Complex-II (Fig. 3E) but measured 17 to 39% decreases in all other mitochondrial protein content with age (Fig. 3C, D, F, G). In females, we measured no change in Complex-III (Fig. 3C), Complex-IV (Fig. 3D), or Complex-I (Fig. 3F) protein, but 33 to 47% increases were evident in the remaining mitochondrial proteins (Fig. 3B, E, G) with age in female mice.

To confirm whether respiratory function differed on a per/mitochondria basis, active respiration data from Fig. 2A was normalized to associated protein levels in Fig. 3 (Fig. 2C). We found that complex-specific respiratory function was lower in females in comparison to aged matched males (2-way ANOVA, *p* < 0.05). Additionally, capacity was reduced as a product of age in all respiratory states measured (2-way ANOVA, *p* < 0.05). Further analysis revealed that this was largely driven by the greater extent of loss in mitochondrial function with age in the female cohort (post hoc, *p* < 0.05). Specifically, normalized Complex-I Active respiration was unchanged in males but was reduced by 49% in females with age. Complex-II Active respiration was non-significantly reduced by 23% but was significantly lower in aged females versus young counterparts by 66%. Finally, Complex-I and II Active respiration was 20% lower in aged males versus young males (not significant) but was 48% lower in aged females versus young females.

### Autophagy-related protein expression in aged muscle

**Fig. 4:**
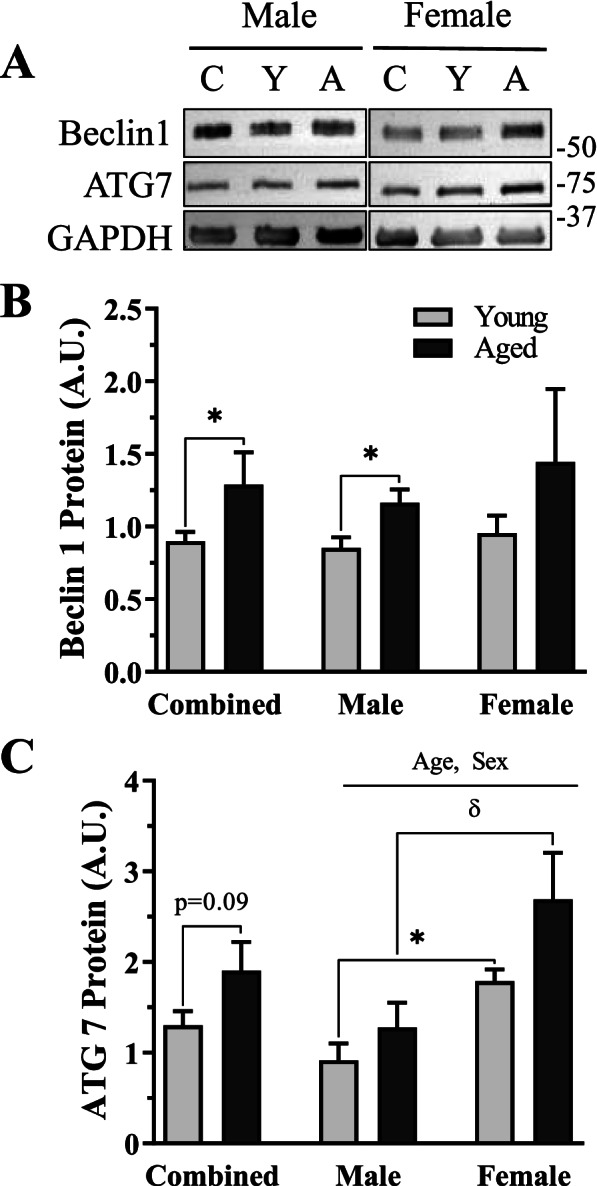
Upstream autophagic proteins in young and age, male and female mice. **A**. Representative western blots for Beclin1 and ATG7. **B** Quantification of Beclin1 protein in combined and sex-separated groups. **C** Quantification of ATG7 protein in combined and sex-separated groups. All values were corrected to GAPDH and are reported as means ± SEM, in A.U. The main effects of 2-way ANOVA are represented on a graph at *p* < 0.05. δ *p* < 0.05, post hoc significance. *p < 0.05, *t*-test between indicated groups. *N* = 10/male group, 8/female group

To evaluate how aging and biological sex affect the autophagy-lysosome system, we measured upstream autophagy proteins in whole muscle quadriceps samples (Fig. 4A–C). In combined-sex groups, aging led to a significant 44% increase in Beclin1 protein (*t*-test, *p* < 0.05, Fig. 4B) and a trending 47% increase in ATG-7 protein (*t*-test, *p* = 0.09, Fig. 4C). When the sexes were analyzed separately, no main or interaction effects were measured in Beclin1 protein (Fig. 4B), but a main effect of both age and sex was found in ATG-7 protein (2-way ANOVA, *p* < 0.05, Fig. 4C), whereby aging and female muscle displayed increased protein expression.

Independent differences between the groups were then examined for these autophagy proteins. Beclin1 protein was significantly increased by 36% in aged males versus young counterparts (*t*-test, *p* < 0.05, Fig. 4B), whereas female mice displayed no age effect (Fig. 4B). ATG-7 protein was unchanged in both sexes independently; however, both young (*t*-test, *p* < 0.05, Fig. 4C) and aged female mice (post hoc, *p* < 0.05, Fig. 4C) contained ~2-fold more ATG-7 protein in comparison to age-matched male mice.

### Autophagosomal-associated protein content in male and female mice with age

**Fig. 5:**
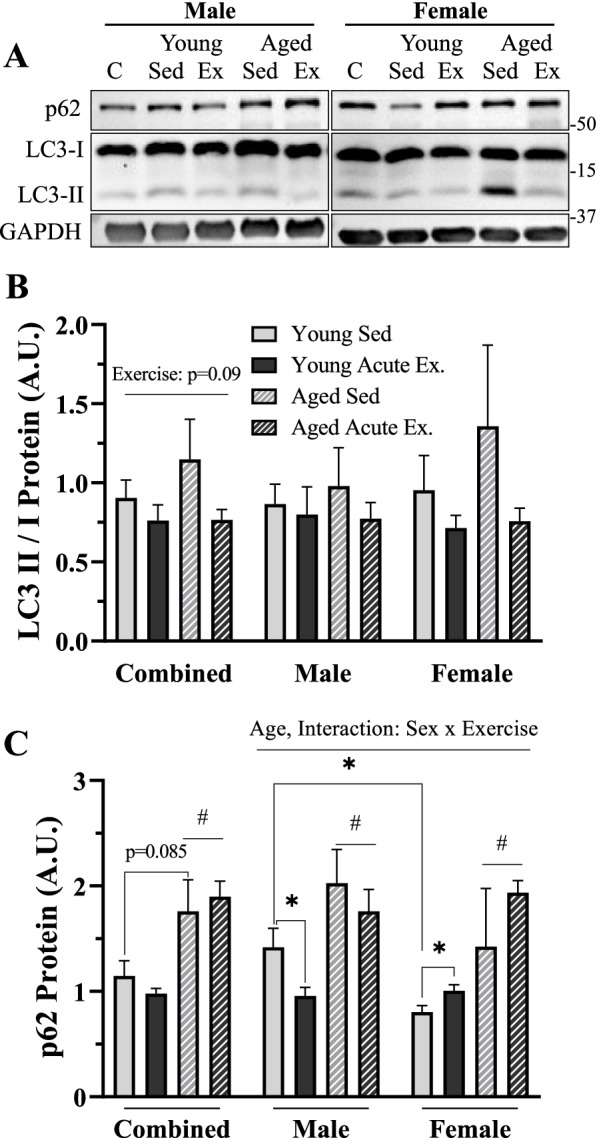
Autophagosomal proteins in sedentary and acute-exercised young and age, male and female mice. **A** Representative western blots for p62, LC3-I, and LC3-II. **B** Quantification of LC3-II/I protein in combined and sex-separated groups. **C** Quantification of p62 protein in combined and sex-separated groups. All values were corrected to GAPDH and are reported as means ± SEM, in A.U. The main effects of a 3-way ANOVA are represented on the graph at *p* < 0.05. The main effects of 2-way ANOVA are represented on a graph at *p* < 0.05. #*p* < 0.05, main effect age. δ *p* < 0.05, post hoc significance. *p < 0.05, *t*-test between indicated groups. *N* = 5/male group, 4/female group

We next wanted to explore how markers of mature autophagosome content were changed in whole muscle samples with age and biological sex in skeletal muscle. We also assessed the impact of exercise in these murine groups. We first measured LC3-II/I as markers of the ratio of mature to immature autophagosomes, respectively. Only minor changes were observed in our combined-group analysis (2-way ANOVA, *p* = 0.09, Fig. 5B), with no main effects or post hoc significance in our sex-separated groups. We observed an overall effect of age on p62 levels in our combined group (2-way ANOVA, *p* < 0.05, Fig. 5C) and a trending 37% increase in p62 protein in our young versus aged sedentary animals (*t*-test, *p* = 0.085, Fig. 5C). In the sex-separated data, a significant main effect of age was observed, along with an interaction between age and acute exercise (3-way ANOVA, *p* < 0.05, Fig. 5C). When we assessed the influence of age and exercise in independent sexes, a main effect of age was evident in both males and females (2-way ANOVA, *p* < 0.05, Fig. 5C). Independent analyses revealed a significant 33% decrease in p62 protein with exercise in young males (*t*-test, *p* < 0.05, Fig. 5C) and a 25% increase with exercise in young females (*t*-test, *p* = 0.05, Fig. 5C).

### Mitophagic protein content in whole muscle of young and aged male and female mice

**Fig. 6:**
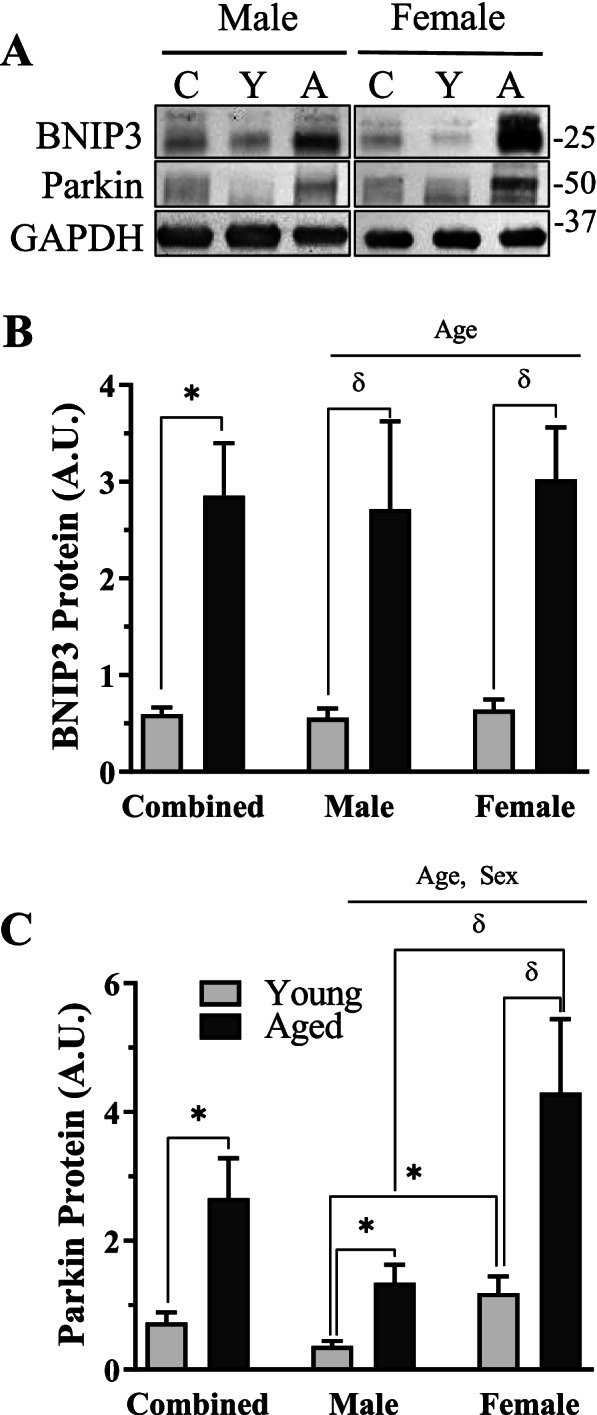
Mitophagy protein content in the muscle and mitochondria in young and aged, male and female mice. **A** Representative western blots for BNIP3 and Parkin in whole muscle samples. **B** Quantification of BNIP3 protein in combined and sex-separated groups. **C** Quantification of Parkin protein in combined and sex-separated groups. Whole muscle values were corrected to GAPDH. Values are reported as means ± SEM, in A.U. The main effects of 2-way ANOVA are represented on a graph at *p* < 0.05. δ *p* < 0.05, post hoc significance. *p < 0.05, *t*-test between indicated groups. *N* = 10/male group, 8/female group

To determine if age and sex impact mitophagy in skeletal muscle, we first probed for the mitophagy markers BNIP3 and Parkin in whole muscle samples (Fig. 6A–C). In the sex-combined group, there were 4.8-fold and 3.6-fold increases in aged muscle BNIP3 and Parkin protein, respectively (*t*-test, *p* < 0.05, Fig. 6B, C). In sex-separated comparisons, a main effect of age was observed in BNIP3 protein (2-way ANOVA, *p* < 0.05, Fig. 6B), and post hoc comparisons revealed similar, significant increases in aged muscle BNIP3 protein vs sex-matched young counterparts (post hoc, *p* < 0.05, Fig. 6B). A main effect of both age and sex was found in Parkin protein, whereby females, both young and old, had more Parkin than their sex-matched, young, counterparts (2-way ANOVA, *p* < 0.05, Fig. 6C). Aging in both sexes led to large increases in Parkin protein (male: *t*-test, *p* < 0.05; female: post hoc, *p* < 0.05; Fig. 6C), suggesting a high capacity for the triggering of mitophagy in aging muscle.

### Lysosomal protein content in young and aged male and female mice

**Fig. 7:**
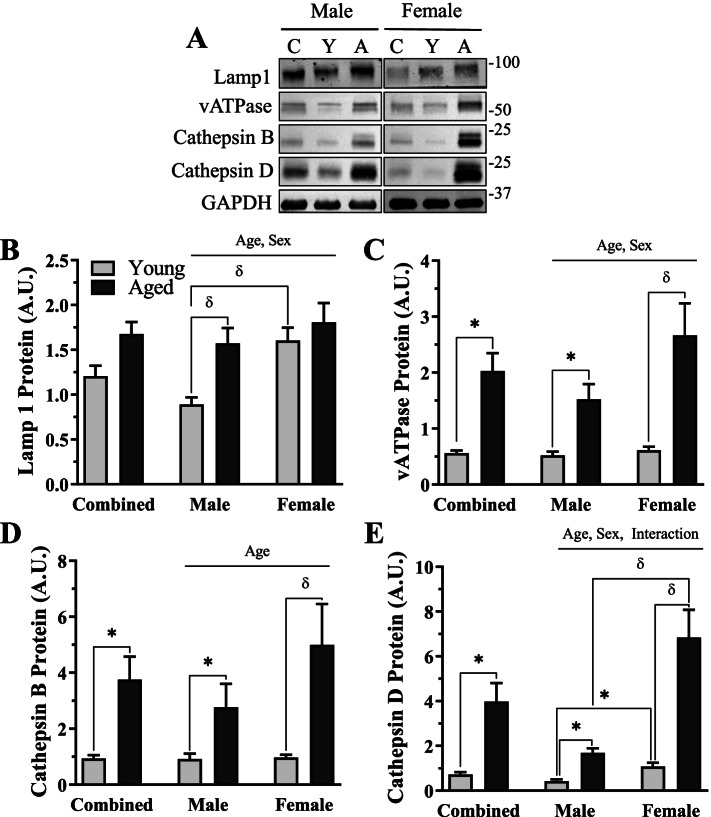
Lysosome proteins in young and age, male and female mice. **A** Representative western blots for Lamp1, vATPase, mature Cathepsin B, and mature Cathepsin D. **B** Quantification of Lamp1 protein in combined and sex-separated groups. **C** Quantification of vATPase protein in combined and sex-separated groups. **D** Quantification of mature Cathepsin B protein in combined and sex-separated groups. **E** Quantification of mature Cathepsin D protein in combined and sex-separated groups. All values were corrected to GAPDH and are reported as means ± SEM, in A.U. The main effects of 2-way ANOVA are represented on a graph at *p* < 0.05. δ *p* < 0.05, post hoc significance. *p < 0.05, *t*-test between indicated groups. *N* = 10/male group, 8/female group

To assess the end-stage of the autophagy pathway, we evaluated lysosomal protein content in our groups (Fig. 7A–E). Lysosome-associated membrane protein 1 (Lamp1) levels were unchanged with age in the sex-combined group. Alternatively, vesicular ATPase (V-ATPase), mature Cathepsin B, and mature Cathepsin D were all upregulated by 3.6-, 4.0-, and 5.5-fold with age, respectively (*t*-test, *p* < 0.05, Fig.7C, D, E). When sex was separated, all lysosomal proteins showed a significant main effect of age (2-way ANOVA, *p* < 0.05, Fig. 7B–E). A main effect of sex was found in Lamp1 (2-way ANOVA, *p* < 0.05, Fig. 7B), vATPase (2-way ANOVA, *p* < 0.05, Fig. 7C), and mature Cathepsin D (2-way ANOVA, *p* < 0.05, Fig. 7E), whereby these proteins were higher in the female mice. An interaction between age and sex was found for mature Cathepsin D protein (two-way ANOVA, *p* < 0.05, Fig. 7E). Independent analyses for each protein confirmed significant 1.8–3.9-fold increases in all measured lysosomal proteins with age in the male mice (*p* < 0.05, Fig. 7B–E). In female mice, significant 4.4–6.5-fold increases were found with age in each lysosome protein (post hoc; *p* < 0.05, Fig. 7C–E), except for Lamp1. We quantified higher Lamp1 (post hoc, *p* < 0.05, Fig. 7B) and mature Cathepsin D (*t*-test, *p* < 0.05, Fig. 7D, E) in young female mice versus young male mice and elevated mature Cathepsin D in aged females compared to aged males (post hoc, *p* < 0.05, Fig. 7E).

**Fig. 8:**
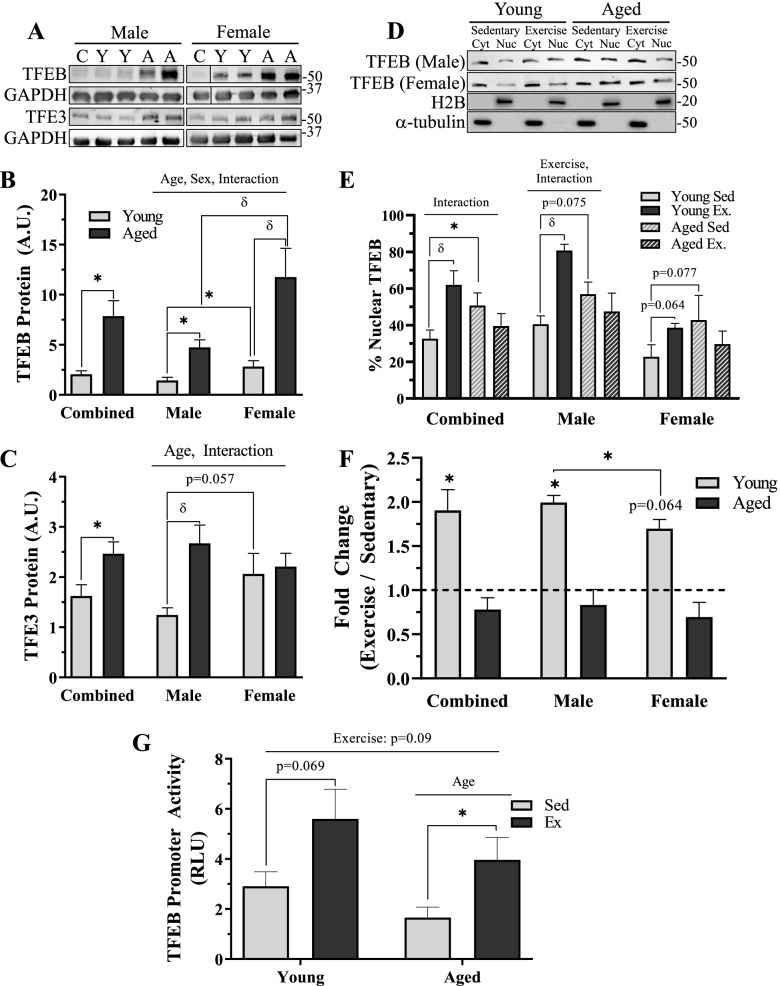
Regulation of lysosome biosynthetic pathways in young and aged, male and female mouse muscle with exercise. **A** Representative western blots for TFEB and TFE3 protein. **B** Quantification of TFEB protein in combined and sex-separated groups. **C** Quantification of TFE3 protein in combined and sex-separated groups. **D** Representative western blots for TFEB protein in nuclear and cytosolic fractions in sedentary and exercised, young and aged, male and female mice. **E** % nuclear TFEB protein in combined and sex-separated male and female mice. **F** Fold-change in nuclear TFEB protein in each group examined. **G** TFEB promoter activity (luciferase; RLU) in young and aged, sedentary, and exercised mice. Values in **B** and **C** were corrected to GAPDH and are reported as means ± SEM. *N* = 10/male group, 8/female group. Line break in representative blot is different sections from the same blot. In **D**–**F**, cytosolic values were corrected to α-tubulin and nuclear values were corrected to H2B and reported as mean ± SEM, in A.U. *N* = 5/male group, 4/female group. In **G**, *N* = 6/group. The main effects of 2-way ANOVA are represented on graph at *p* < 0.05. δ *p* < 0.05, post hoc significance. *p < 0.05, *t*-test between indicated groups

We also measured the protein levels of TFEB and TFE3, transcription factors that control the autophagy-lysosome pathway (Fig. 8A–C). TFEB was 3.8-fold greater with age in the sex-combined analysis (*t*-test, *p* < 0.05, Fig. 8B). In the sex-separated analyses, TFEB protein exhibited main effects of age and sex, and an interaction existed between these variables (2-way ANOVA, *p* < 0.05, Fig. 8B). This protein was greater in aged, compared to young muscle, and muscle from females exhibited higher TFEB levels than in male counterparts, and this was amplified further with age. Specifically, TFEB protein was 1.8-fold greater in young females (*t*-test, *p* < 0.05, Fig. 8B) and 2.5-fold greater in aged females (*t*-test, *p* < 0.05, Fig. 8B) when compared to age-matched male counterparts. Compared to young, sex-matched animals, TFEB protein was 3.3-fold greater in aged males (*t*-test, *p* < 0.05, Fig. 8B) and 4.2-fold higher in aged females (post hoc, *p* < 0.05, Fig. 8B). Conversely, TFE3 was 1.5-fold greater in our sex-combined analysis (*t*-test, *p* < 0.05, Fig. 8C). In the sex-separated analyses, TFE3 protein exhibited main effects of age and interaction between age and sex (2-way ANOVA, *p* < 0.05, Fig. 8C). As such, TFE3 protein was increased 3.3-fold with age in male mice (post hoc, *p* < 0.05, Fig. 8C), an effect not seen in females. A trending sex difference was also measured in TFE3 protein, whereby young females contained 66% more than young males (*t*-test, *p* = 0.057, Fig. 8C).

**Fig. 9:**
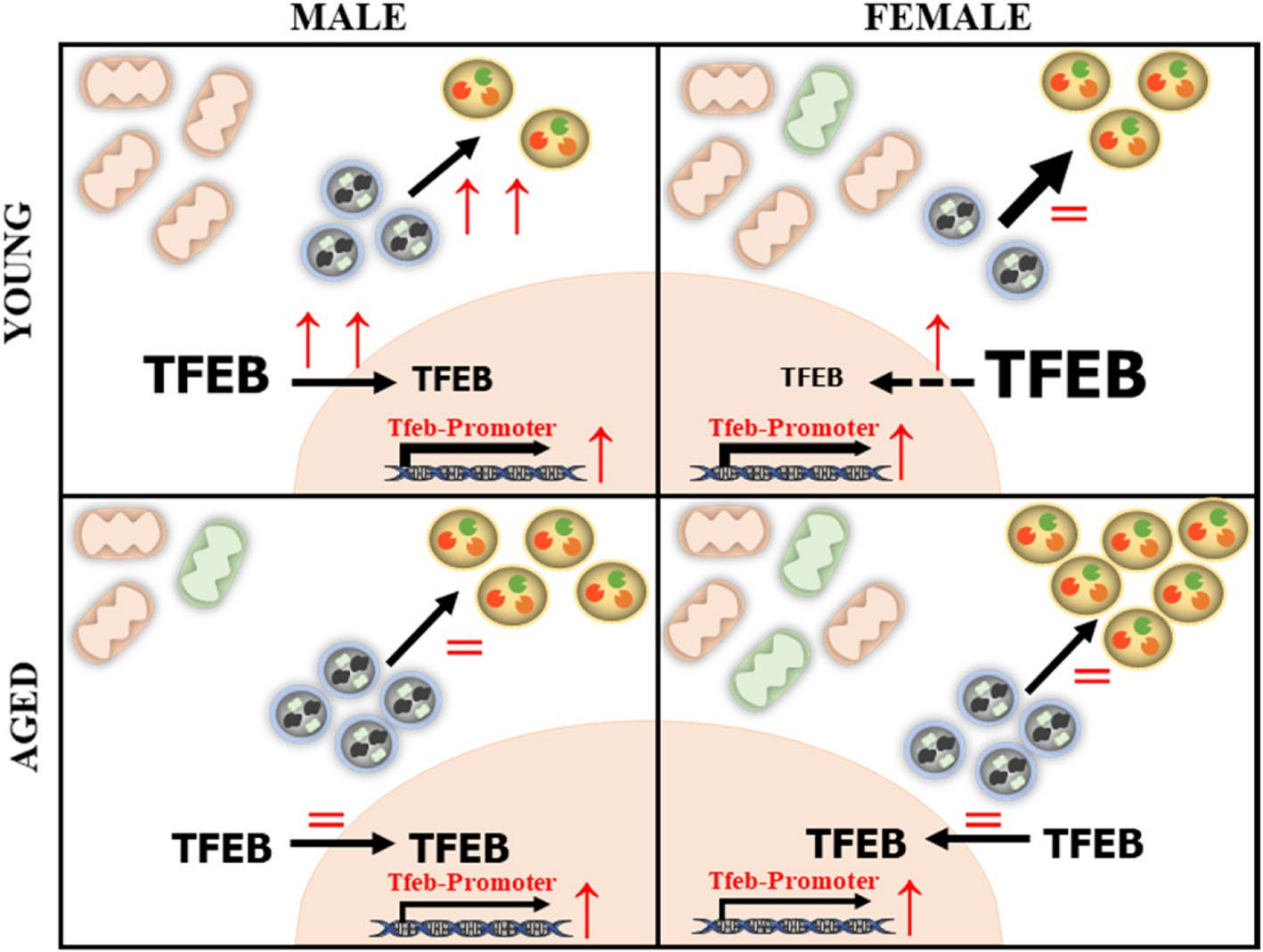
Summary of sex differences in the skeletal muscle of young and aged mice and the response to acute exhaustive exercise. Muscle from young males (top left panel) contains fewer mitochondria of higher functionality, less lysosomes, and their transcriptional regulator TFEB in comparison to young females (top right panel). With aging, there are decrements in mitochondrial content and function in males (bottom left). Alternatively, in females, mitochondrial content was relatively unchanged, although function was reduced. These changes were associated with a greater upregulation in the autophagy-lysosome system in females with age. With exercise, there was an induction of autophagy and nuclear TFEB in young males that was not present in young females or aged animals of either sex. Overall, exercise was capable of enhancing TFEB promoter activity. Font size represents the relative amount of TFEB; thickness of black arrows represents the relative contribution of the autophagy-lysosome system at baseline; red front dictates change with exercise (↑ represents increase, = represents no change). Orange mitochondria are healthy, and green are unhealthy

### Influence of exercise on lysosome biosynthetic pathway

We wished to explore whether exercise could activate lysosome biosynthesis pathways in both young and aged, male and female muscle (Fig. 8D–G)Thus, we measured the percent of nuclear TFEB protein in all groups. Muscle from aged sedentary, sex-combined mice contained 18% more nuclear TFEB (*t*-test, *p* < 0.05, Fig. 8E). In this sex-combined analysis, there was an interaction between age and exercise (2-way ANOVA, *p* < 0.05, Fig. 8E). Specifically, following the cessation of exercise, nuclear TFEB was increased by 30% in the young sex-combined cohort (post hoc, *p* < 0.05, Fig. 8E), which was not evident in the muscle from aged animals. In the sex-separated analysis, aged male and female muscle appeared to possess approximately 20% higher basal levels of TFEB in the nucleus, compared to young counterparts (*t*-test, *p* = 0.075 and *p* = 0.077, male and female, respectively, Fig. 8E). In response to exercise, young male mice enhanced nuclear TFEB by 40% (post hoc, *p* < 0.05, Fig. 8E), which was modestly greater than the effect observed in females (*t*-test, *p* = 0.064, Fig. 8E; *t*-test, *p* < 0.05, Fig. 8F).

We also utilized a TFEB-luciferase promoter activity assay to determine whether exercise stimulates TFEB transcriptional activity. This analysis could only be completed in sex-combined groups. Overall, there was a trending main effect of increased promoter activity with exercise (2-way ANOVA, *p* = 0.09, Fig. 8G). There was also a main effect of age (2-way ANOVA, *p* < 0.05, Fig. 8G), whereby TFEB promoter activity was reduced with age. A trending 1.9-fold increase in TFEB promoter activity in young mice was observed (*t*-test, *p* = 0.069, Fig. 8G), whereas a significant 2.5-fold increase was measured in the aged cohort as a result of acute exercise (*t*-test, *p* < 0.05, Fig. 8G).

## Discussion

It is well established that regular exercise can counteract the progressive loss of muscle mass and function associated with advancing age [36–38]. This is partially through upregulating the production of healthy cellular components, such as mitochondria, and stimulating the removal of dysfunctional constituents through the autophagy-lysosomes system (i.e., mitophagy) [20, 39]. Previous reports from our group have indicated that acute exercise initiates lysosome biogenesis [26] and that chronic muscle activity elevates lysosome protein content [30, 40] in young, healthy muscle. These changes are likely to support an enhanced capacity for proteolysis. However, no studies to date have examined the regulation of autophagy and lysosome biosynthesis in sedentary or exercised aged muscle with a focus on the influence of biological sex. To this end, the present study explores how biological sex influences the autophagy-lysosome system in young and aged skeletal muscle. Furthermore, we wanted to examine whether acute exercise could upregulate lysosomal synthetic pathways in aged muscle and whether the effect was sex-dependent.

To address these aims, we utilized young (4–6 months) and aged (22–24 months), male and female C57BL/6 mice, which were either sedentary or exercised acutely. We confirmed that female mice have a smaller muscle mass, as previously reported [41], which may be due in part to a higher abundance of smaller, type I, muscle fibers in females [42–45]. Although it has been previously reported that female rodents have greater loss of muscle mass with hindlimb unloading [32, 33, 46], our data show that this sexual dimorphism does not occur with aging. Specifically, the greater reductions in muscle mass that the female cohort display with age are largely due to the smaller age-related gain in body weight.

Our data also confirm a multitude of reports showing that aging leads to decrements in endurance capacity [20, 39]. We further report that young female mice have greater exercise capacity than their male counterparts. As post-exercise lactate levels were similar amongst all groups, we can eliminate the possibility that males and females were not similarly exhausted. Thus, the longer distance to exhaustion in young female mice may be due to the greater abundance of mitochondrial proteins that we measured in our female cohort, and their role in mediating endurance capacity. Supportively, others have found that young female muscle contains greater mitochondrial content [34, 47, 48] and is more fatigue-resistant [49–51]. However, our analysis revealed a greater decline in mitochondrial function in female muscle, compared to males, with age. This may account for the greater reduction in exercise tolerance in our female cohort and may serve as a stimulus for the biogenesis of autophagy and lysosomal proteins responsible for organelle clearance.

A connection between mitochondrial respiratory deficits and the autophagy-lysosome system does exist in various contexts. For example, deletion of the mitochondrial fusion mediator, OPA1, in muscle enhances autophagy and lysosome components, while also elevating autophagic flux [52]. Similarly, others have shown that loss of mitochondrial function either by deletion of integral proteins or by treatment of cells with mitochondrial inhibitors and uncouplers impairs lysosome function [53]. Mechanistically, loss of oxidative capacity serves to upregulate TFEB levels and subsequently autophagic and lysosomal proteins [54]. We have also shown that in both denervation-induced muscle atrophy [55] and aging muscle [22], mitochondrial functional impairments are correlated with a greater abundance of autophagy and lysosome proteins, which is in part driven by greater TFEB protein. It seems apparent that a regulatory network exists, whereby impaired mitochondria signal toward the autophagy-lysosome system, likely with a purpose of degrading the accumulating damaged organelles and proteins. Thus, it is plausible that the modest deficits in mitochondrial function that we report in the young female mice contribute to a greater reliance of the autophagy-lysosome system. Supportively, we show that this cohort has more of the LC3-maturation protein, ATG7. In conjunction with a less p62 protein, which is degraded by lysosome proteolysis, this suggests a higher basal autophagic breakdown in our young female, compared to our young male cohort, similar to a previous report [56]. As such, the greater abundance of lysosome proteins, which may be due to a higher level of the transcription factors TFEB and TFE3, in young females likely serves to support this elevated basal autophagy.

Similarly, with aging, the loss of mitochondrial function in both sexes may explain the considerable upregulation of the upstream autophagy proteins Beclin 1 and ATG7, alike previous reports [22, 23]. We also found that aged muscle contains a higher abundance of p62 and total LC3 (data not shown), indicative of greater autophagic signaling with age. Finally, we believe that the more pronounced loss in mitochondrial function within aging female muscle may augment this pathway, explaining the relatively greater increase in autophagy-lysosome components with age. It is not clear whether the mere abundance of lysosomal proteins reflects a functional improvement with both age and sex. For example, in muscle from aged rodents and humans, the presence of non-digestible lysosomal content, termed lipofuscin, is apparent [23, 57]. Thus, the greater abundance of lysosome protein reported previously [22, 58] and in the present study may be evidence of dysfunctional lysosomes that may limit the capacity for autophagic and mitophagic breakdown. Alternatively, this could be explained by the greater levels of both whole muscle TFEB and TFE3 protein, as well as nuclear TFEB protein, in the muscle from aged animals in this study.

With an interest in how these changes may translate to mitochondrial degradation through mitophagy, we assessed levels of proteins involved in targeting these organelles for digestion at the lysosomes. In whole muscle samples, the age-induced increases in both BNIP3 and Parkin imply greater mitophagy-targeting. In addition, the greater levels of BNIP3 in young females, along with the more pronounced increase in females with age, suggest a divergent regulation of mitophagy-targeting mechanisms in male and female muscle. We have previously reported that basal mitophagy flux is enhanced in aged muscle [22, 24] but in contrast to young muscle, that it is unresponsive to the stress of acute exercise. Future work will be required to determine whether the exercise effect on mitophagy is dependent on sex.

Acute endurance exercise is a stimulus for autophagosomal turnover in young muscle [19, 59, 60]. Thus, we wanted to examine if sex and/or age influence this response. In response to acute exercise, reductions in p62 were observed in young male mice, suggesting an exercise-induced increased in autophagic clearance. In contrast, increases in p62 protein were observed in age-matched females. This implies that autophagosomal breakdown is stimulated exclusively in young male muscle with acute exercise. Since female muscle appears to have accelerated basal autophagy, as noted above, exercise may be insufficient to further augment this degradation pathway. In aged muscle, these exercise effects observed in young males were not apparent, regardless of sex, suggesting that the effects of exercise on autophagy are both sex- and age-dependent. It should be noted that although our measures of autophagy do not directly indicate true autophagic flux, we have recently reported that reductions in p62 in skeletal muscle are strongly correlated with elevated autophagy flux [61]. Thus, we feel our interpretation of autophagic breakdown at baseline and with exercise is warranted.

In contrast to the effect of aging, our work in the past has uncovered that chronic contractile activity, as a model of endurance exercise training, also leads to an upregulation of lysosome content in young healthy muscle [22, 30, 40]. This is due to acute exercise inductions in the transcriptional activation of TFEB, as well as its enhanced nuclear localization [26]. When we analyzed this response in males versus females, the relative shift of TFEB to the nucleus was greater in the male cohort. This finding matches that of the exercise-induced change in autophagy found exclusively in the muscle of young male animals. Cumulatively, these findings imply that the autophagy-lysosome system is more responsive to exercise in young male, compared to female muscle. We also wanted to examine these mechanisms in aged muscle, as increasing lysosomal content through exercise could in theory enhance the capacity for autophagic/mitophagic degradation. We hypothesized that the muscle from aged animals would exhibit a blunted lysosome biosynthetic pathway due to attenuated kinase signaling [62, 63]. Supportively, an upregulation of nuclear TFEB protein post-exercise was only found in young animals. However, although aging led to an overall decrease in TFEB transcription as measured by promoter-reporter activity level, exercise was able to upregulate it to the level observed in young muscle. Thus, even though aging blunts aspects of autophagosomal turnover and lysosome biosynthesis with acute exercise, it enhances TFEB transcription, which, if repeated over time, may serve to re-establish lysosome function and homeostasis.

## Conclusions

Our studies reveal that aging and exercise have differential effects on the autophagy-lysosome system in skeletal muscle, which is dependent on biological sex (Fig. 9). First, we show that in female mice, there is a greater abundance of mitochondrial, autophagy, and lysosome proteins. Second, we observed that female mice have a greater index of basal skeletal muscle autophagosome clearance than male mice. We also demonstrate that acute exercise stimulates autophagosome turnover and lysosome biogenesis in young males, but not in young females or in aged animals from either sex. Finally, and most importantly, exercise was able to activate TFEB promoter activity regardless of age, which can promote lysosome biogenesis. This finding provides merit to further explore the vitality of exercise in re-establishing autophagy-lysosome homeostasis in aged muscle. In addition, more work is required to delineate the impact of biological sex on mechanisms that regulate mitochondrial-lysosome interactions in skeletal muscle.

## Data Availability

All raw data used to generate the figures are available upon request from Dr. David A. Hood (dhood@yorku.ca).

## Abbreviations

RLU: Relative light units
OXPHOS: Oxidative phosphorylation
ATG7: Autophagy-related gene 7
p62: Sequestosome 1
LC3: Microtubule-associated protein 1A/1B-light chain 3
Lamp 1: Lysosome-associated membrane protein 1
V-ATPase B1/2: Vesicular ATPase B1/2
TFEB: Transcription factor EB
TFE3: Transcription factor E3
H2B: Histone 2 B
TA: Tibialis anterior
Sol: Soleus
ETC: Electron transport chain
